# Supplementary material for: Common and divergent gene regulatory networks control injury-induced and developmental neurogenesis in zebrafish retina
Source: Nat Commun. 2023 Dec 20;14:8477. doi: 10.1038/s41467-023-44142-w (PMC10733277; doi:10.1038/s41467-023-44142-w)
Supplement: Supplementary file 1 — Supplementary Information [file 41467_2023_44142_MOESM1_ESM.docx]

**Supplementary information**

**Common and divergent gene regulatory networks control injury-induced and developmental neurogenesis in zebrafish retina**


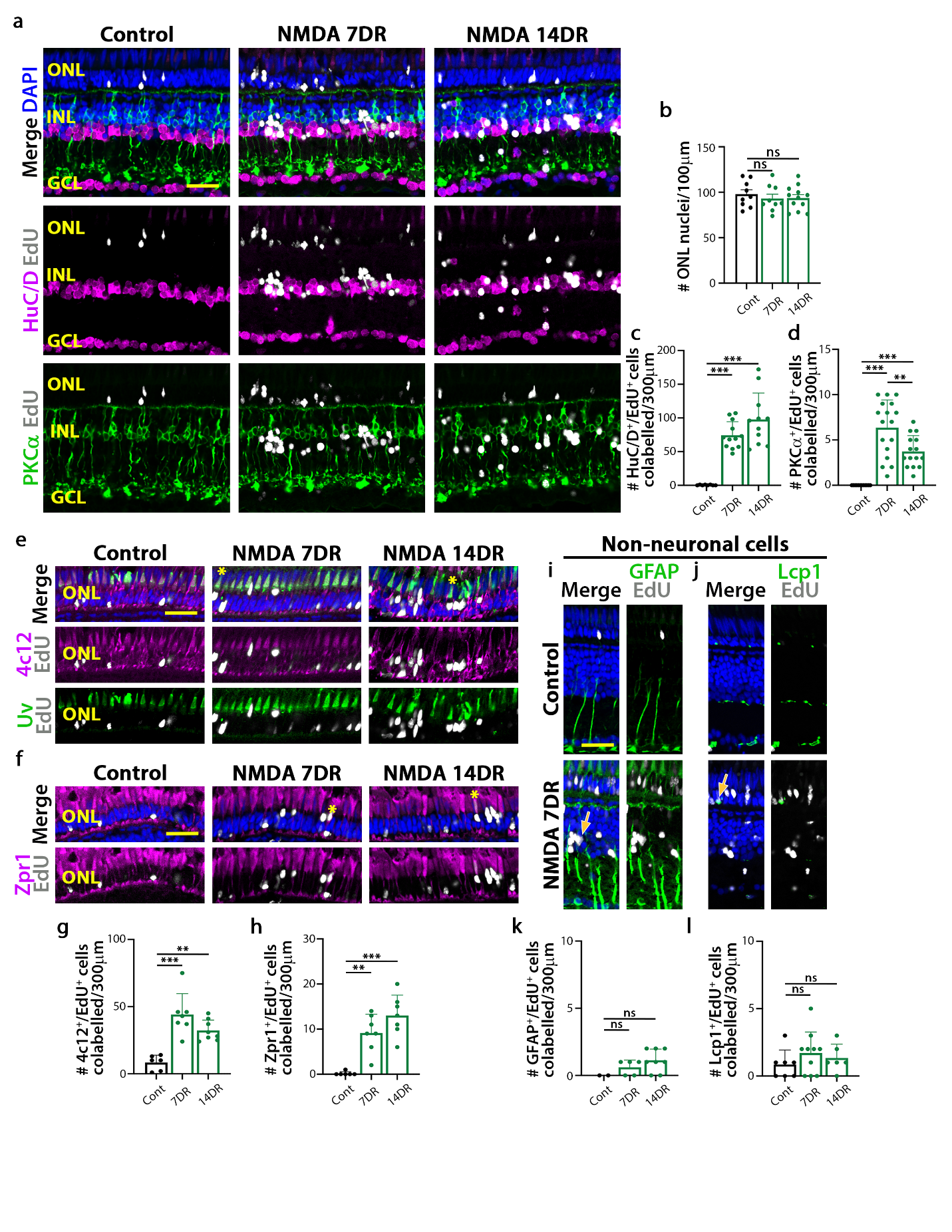


**Supplemental Figure 1: Regeneration of retinal neurons following NMDA damage.** (a)  EdU-labeling in retinas prior to NMDA damage (Control) and 7 and 14 days following 96 hours of NMDA damage. Retinas were immunostained for EdU, HuC/D, and PKCα and counterstained with DAPI.

(b) Quantification of the number of DAPI-labeled nuclei in the INL. Control (Cont) n=9, 7 days recovery (DR) n=9, 14DR n=12.

(c) Quantification of the number of cells colabeled for EdU and HuC/D. Control (Cont) n=8, 7DR n=12, 14DR n=11. Three independent experiments.

(d) Quantification of the number of cells colabeled for EdU and PKCα. Control (Cont) n=12, 7DR n=17, 14DR n=15. Three independent experiments.

(e) EdU-labeling in retinas prior to NMDA damage (Control) and 7 and 14 days following 96 hours of NMDA damage. Retinas were immunostained for EdU, 4c12, and UV opsin and counterstained with DAPI.

(f) EdU-labeling in retinas at 7 and 14 days following 96 hours of NMDA damage. Retinas were immunostained for EdU and Zpr1 and counterstained with DAPI.

(g) Quantification of the number of cells colabeled for EdU and 4c12. Control (Cont) n=6, 7DR n=7, 14DR n=8. Two independent experiments.

(h) Quantification of the number of cells colabeled for EdU and Zpr1. Control (Cont) n=6, 7DR n=7, 14DR n=7. Two independent experiments.

(i) EdU-labeling in retinas prior to NMDA damage (Control) and 7 days following 96 hours of NMDA damage. Retinas were immunostained for EdU and GFAP and counterstained with DAPI.

(j) EdU-labeling in retinas prior to NMDA damage (Control) and 7 days following 96 hours of NMDA damage. Retinas were immunostained for EdU and Lcp1 and counterstained with DAPI.

(k) Quantification of the number of cells colabeled for EdU and GFAP. Control (Cont) n=2, 7DR n=5, 14DR n=8. One independent experiment.

(l) Quantification of the number of cells colabeled for EdU and Lcp1. Control (Cont) n=7, 7DR n=10, 14DR n=6. Two independent experiments.

Scale bars in a, e, f, i, and j are 20μm. b, c, d, g, h, k, and l data are presented as mean values +/- SEM. Asterisks indicate statistically significant differences between the indicated groups (***p* ≤ 0.01, ****p* ≤ 0.001). Source data are provided as a Source Data file 1.


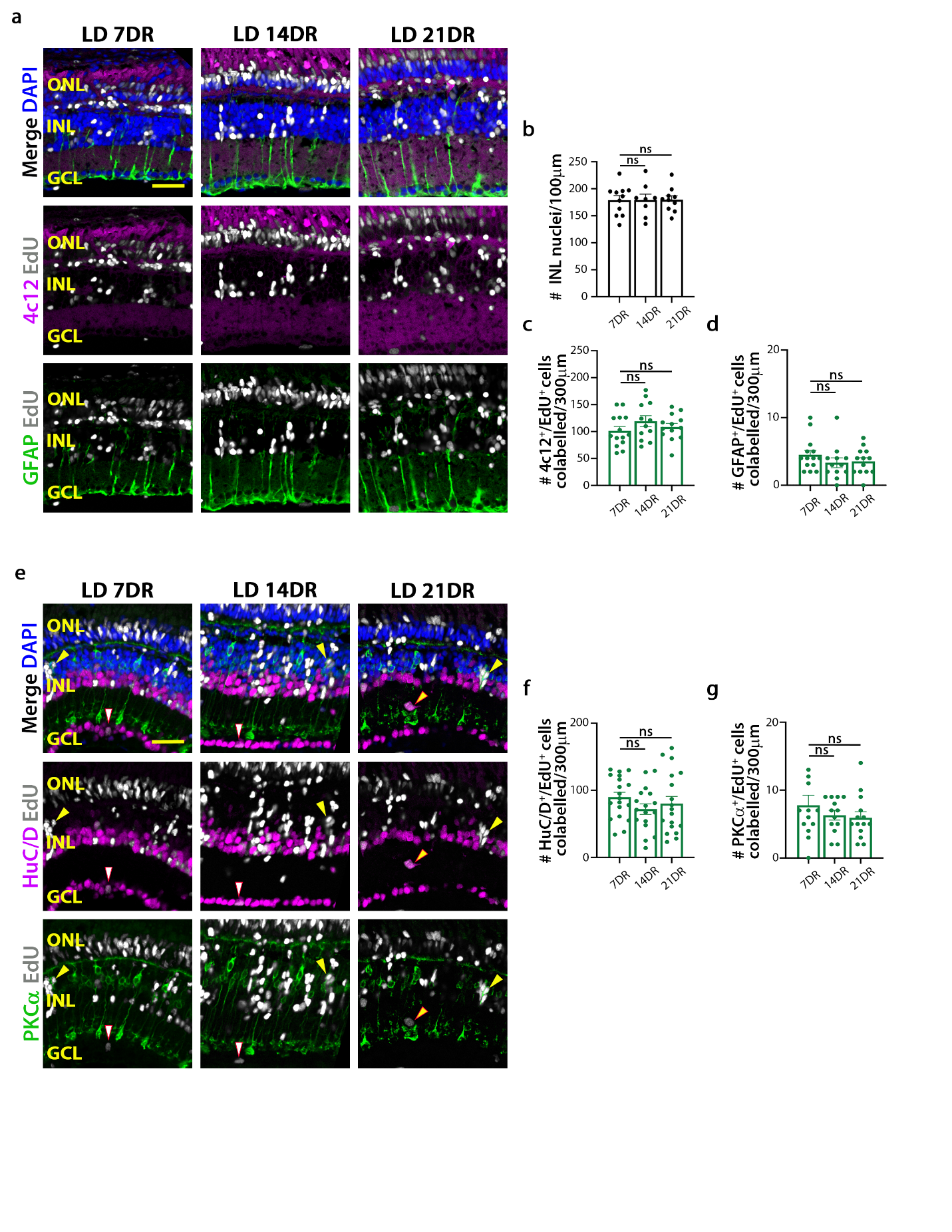


**Supplemental Figure 2: Regeneration of retinal neurons following constant light damage.**

(a) EdU-labeling in retinas at 7, 14, and 21 days following 96 hours of constant light. Retinas were immunostained for EdU, 4c12, and GFAP and counterstained with DAPI.

(b) Quantification of the number of DAPI-labeled nuclei in the INL. n=11; 8; 10 for 7DR; 14DR; 21DR, respectively. 7DR. Two independent experiments.

(c) Quantification of the number of cells colabeled for EdU and 4c12.  n=14; 12; 13 for 7DR; 14DR; 21DR, respectively.

(d) Quantification of the number of cells colabeled for EdU and GFAP.  n=14; 12; 13 for 7DR; 14DR; 21DR, respectively.

(e) EdU-labeling in retinas at 7, 14, and 21 days following 96 hours of constant light. Retinas were immunostained for EdU, HuC/D, and PKCα and counterstained with DAPI. (f) Quantification of the number of cells colabeled for EdU and HuC/D.  n=19; 17; 18 for 7DR; 14DR; 21DR, respectively.

(g) Quantification of the number of cells colabeled for EdU and PKCα. n=13; 13; 14 for 7DR; 14DR; 21DR, respectively.

Scale bars in a and e are 20μm. b, c, d, f, and g data are presented as mean values +/- SEM (ns, not significant). Source data are provided as a Source Data file 1.


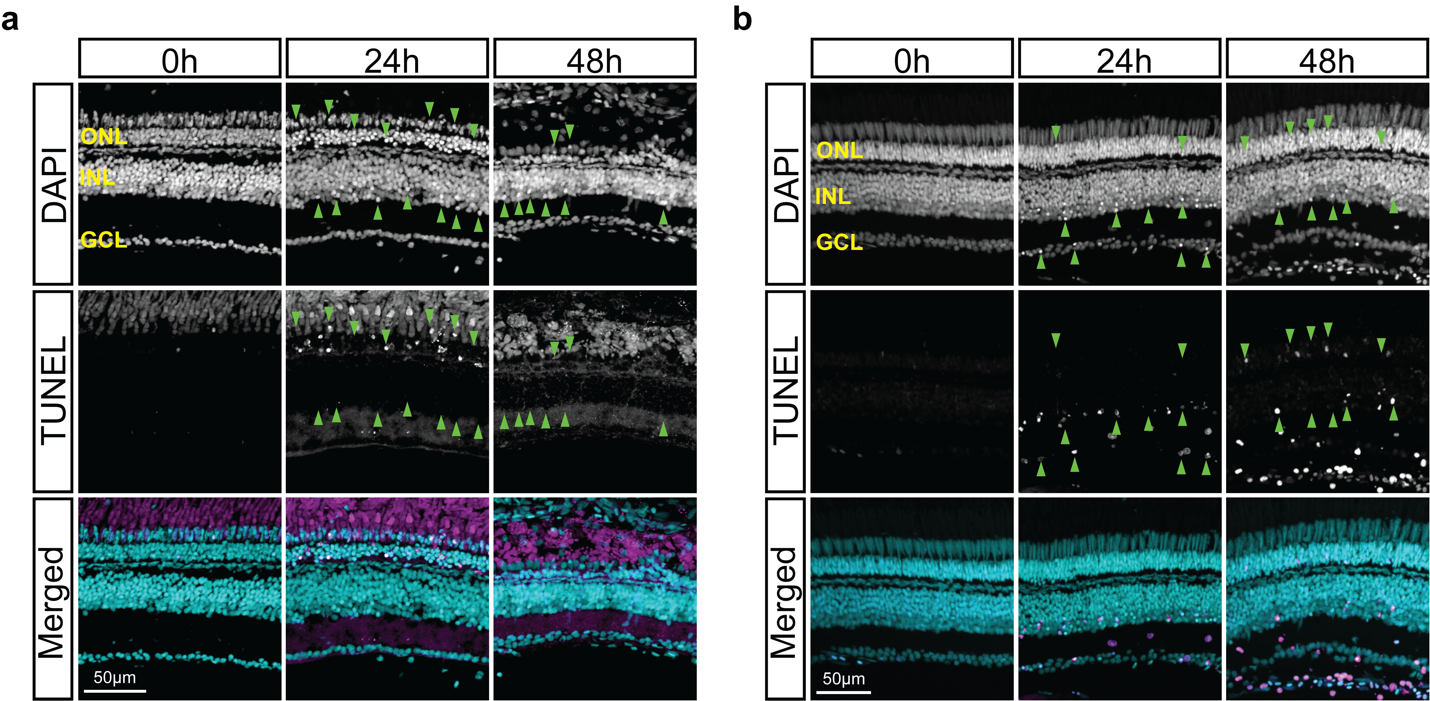


**Supplemental Figure 3: Indirect neuronal cell death is observed following constant light damage and NMDA excitotoxicity.**

(a) Comparison of undamaged and light-damaged retinas at 24 and 48h. Pyknotic nuclei (green arrowheads) and TUNEL-positive cells are observed in the ONL, as expected given the photoreceptor loss due to phototoxicity. Additionally, pyknotic nuclei are observed within the basal portion of the INL beginning at 24h of light damage. Not all pyknotic nuclei in the ONL are TUNEL-positive; and none of the pyknotic nuclei in the INL are TUNEL-positive. At 48h, most of the photoreceptors have died, though some pyknotic nuclei are still observed within the ONL and the INL, though they are not TUNEL-positive.

(b) Comparison of undamaged and NMDA-damaged retinas at 24 and 48h. Pyknotic nuclei (green arrowheads) and TUNEL-positive cells are observed in the GLC and basal portion of the INL, as expected in this model. Additionally, pyknotic nuclei are observed within the ONL beginning at 24h, though they are not TUNEL-positive. At 48h both pyknotic and TUNEL-positive cells are present within the ONL and INL, and TUNEL-positive cells also appear in the ONL.


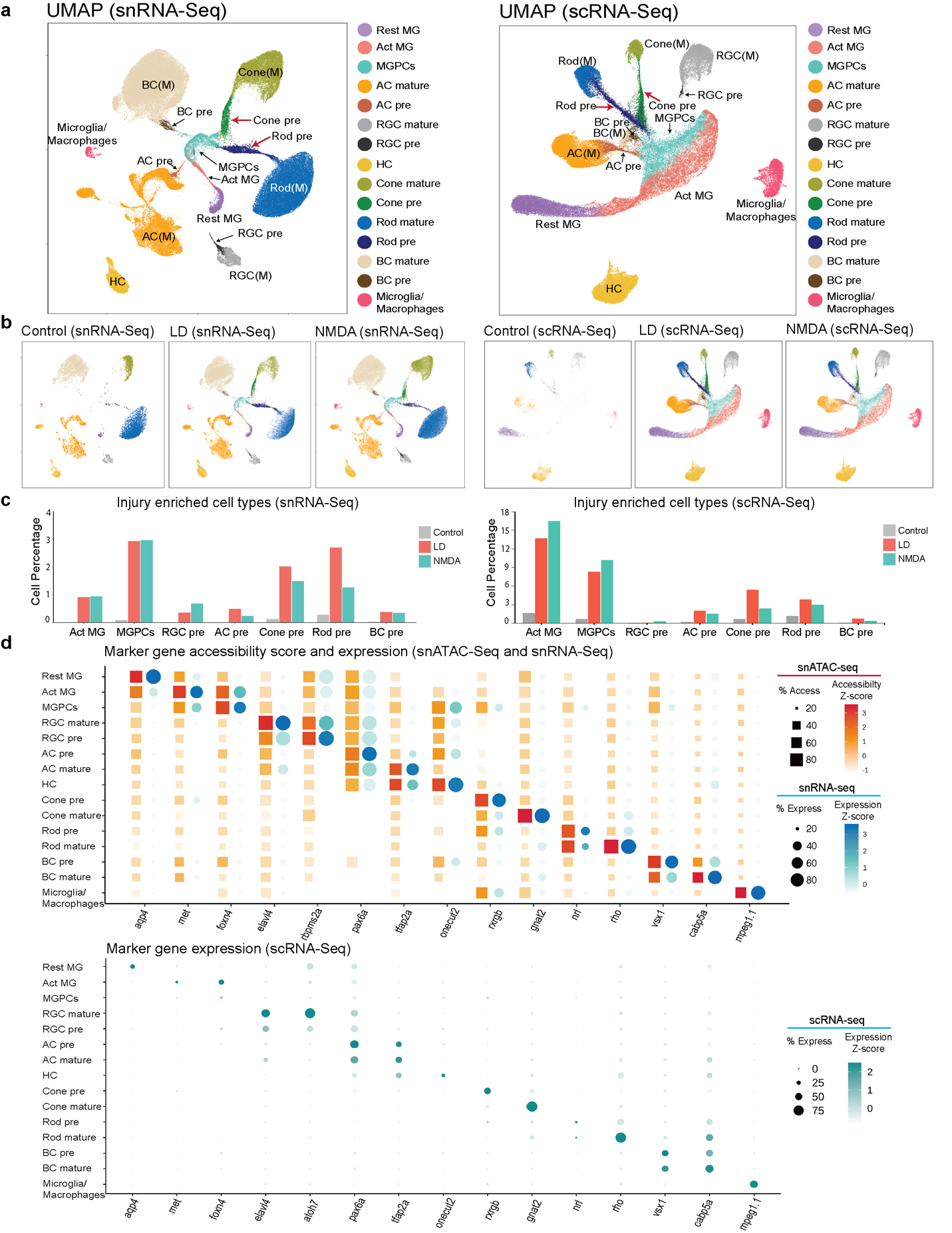


**Supplemental Figure 4:  Overview of single-cell sequencing datasets from LD and NMDA injury models.**

(a) Combined UMAP plots showing the cells in LD and NMDA datasets (left: snRNA-Seq, right: scRNA-Seq). Each point represents an individual cell and is colored by its corresponding cell type.

(b) UMAP plots (left:snRNA-Seq, right:scRNA-Seq) showing the cells separated by each condition for both snRNA-Seq and scRNA-Seq datasets.

(c) The bar plot (left:snRNA-Seq, right:scRNA-Seq) indicates Act MG, MGPCs and newly-born neuron precursors are both enriched in the two injury models compared to the control dataset. The y-axis indicates the cell ratio at each time point. Bars are colored by conditions.

(d) Examples of mRNA levels and chromatin accessibility (up panel:snRNA-Seq and snATAC-seq, down panel:scRNA-Seq) for selected cell-type-specific genes.


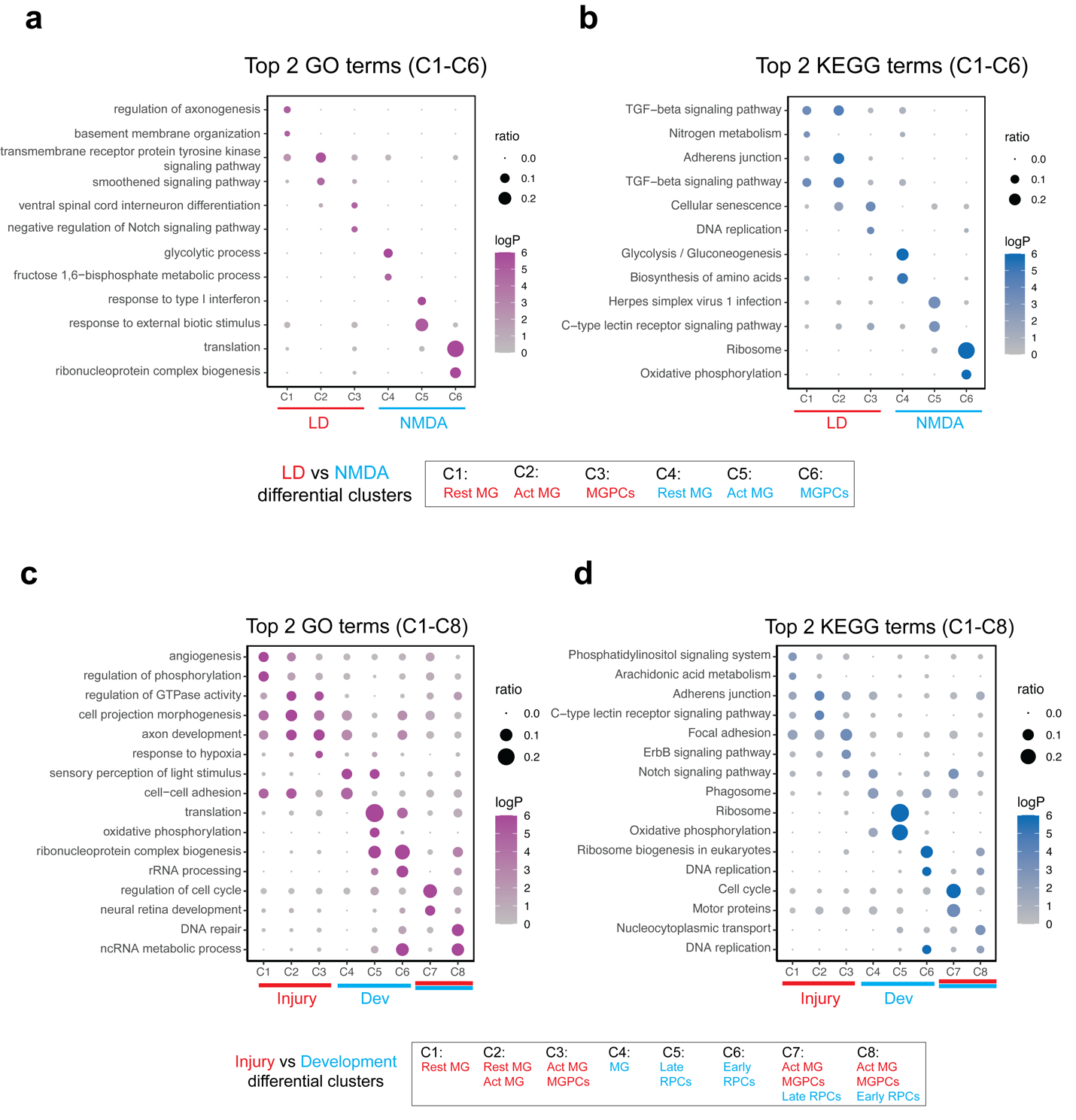


**Supplemental Figure 5: Enriched GO and KEGG terms in LD and NMDA, and injury and development.**

(a,b) Dot plots showing the most highly enriched 2 GO and KEGG terms enriched for differential gene clusters between LD and NMDA models. The x-axis indicates the clusters, and the y-axis indicates the GO or KEGG terms, the size of the dot represents the gene ratio and the color represents the negative log-transformed p-values.

(c,d) Dot plots showing the most highly enriched 2 GO and KEGG terms enriched for differential gene clusters between injury and development models. The x-axis indicates the clusters, and the y-axis indicates the GO or KEGG terms, the size of the dot represents the gene ratio and the color represents the negative log-transformed p-values.


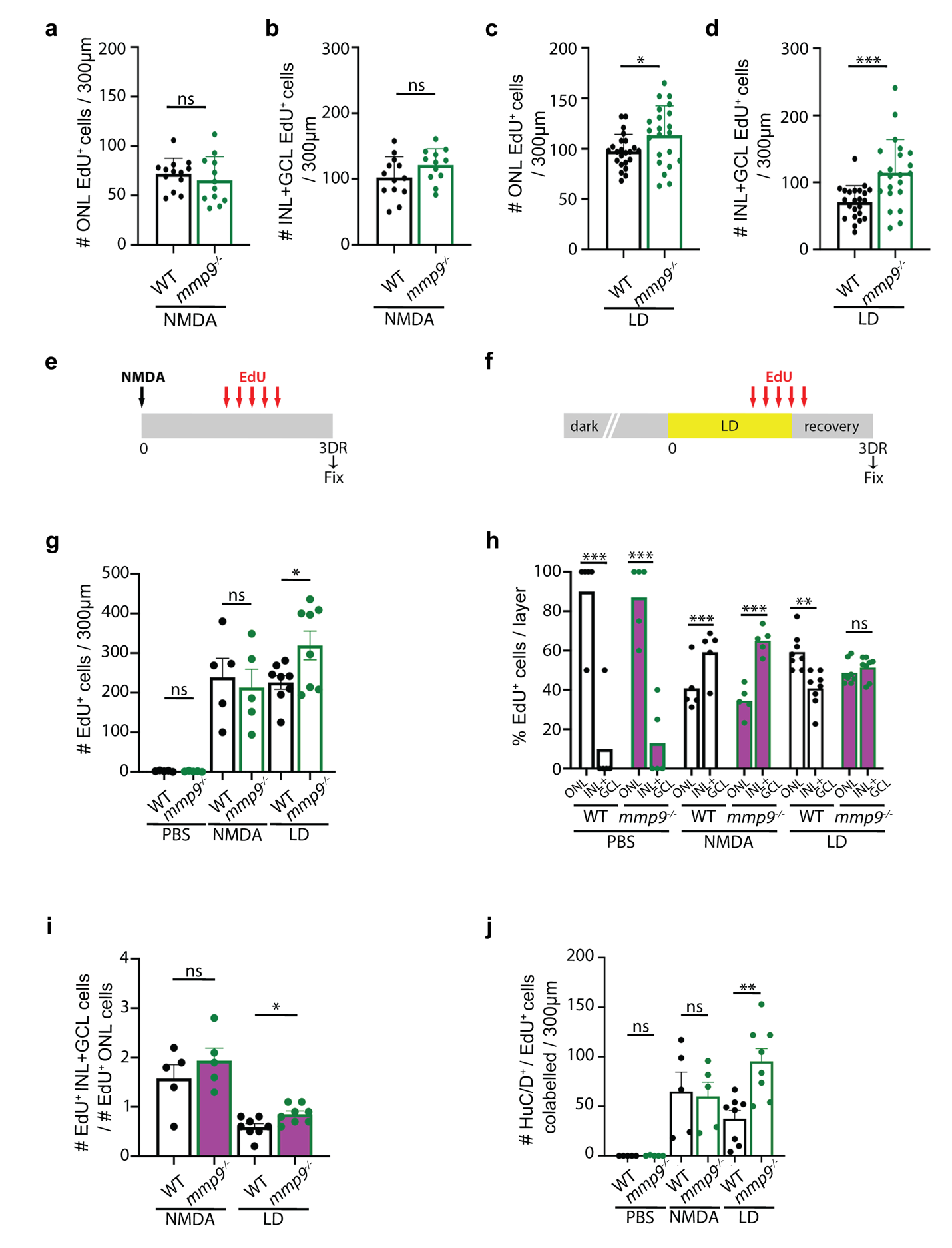


**Supplemental Figure 6: Mmp9 regulates regeneration of HuC/D-labeled amacrine and ganglion cells**.

(a) Quantification of the number of EdU-labeled ONL cells in NMDA-injected wild-type and *mmp9* mutant retinas at 7DR. n=13 and 12 for WT and *mmp9* mutants, respectively. Three independent experiments.

(b) Quantification of the number of EdU-labeled INL+GCL cells in NMDA-injected wild-type and *mmp9* mutant retinas at 7DR. n=13 and 12 for WT and *mmp9* mutants, respectively. Three independent experiments.

(c) Quantification of the number of EdU-labeled ONL cells in light-damaged wild-type and *mmp9* mutant retinas at 7DR. n=23 and 22 for WT and *mmp9* mutants, respectively. Three independent experiments.

(d) Quantification of the number of EdU-labeled INL+GCL cells in light-damaged wild-type and *mmp9* mutant retinas at 7 DR. n=23 and 22 for WT and *mmp9* mutants, respectively. Three independent experiments.

(e) Schematic of NMDA-induced damage experiment.

(f) Schematic of light-induced damage experiment.

(g) Quantification of the number of EdU-labeled cells in all three retinal layers in wild-type and *mmp9* mutants following either PBS injection (n=5 for WT and n=5 *mmp9* mutant), NMDA damage (n=5 for WT and n=5 *mmp9* mutant), or light damage (n=8 for WT and n=8 *mmp9* mutant) at 3DR.

(h) The percentage of EdU-positive cells in the ONL versus the INL+GCL is plotted for wild-type and *mmp9* mutant retinas after PBS injection (n=5 for wt and n=5 *mmp9* mutant), NMDA damage (n=5 for wt and n=5 *mmp9* mutant), and light damage (n=8 for WT and n=8 *mmp9* mutant) at 3DR.

(i) The ratio of EdU-positive INL+GCL cells to EdU-positive ONL cells is plotted for wild-type and *mmp9* mutant fish following either NMDA damage (n=5 for WT and n=5 *mmp9* mutant) or light damage (n=8 for WT and n=8 *mmp9* mutant) at 3 DR.

(j) Quantification of the number of cells colabeled for EdU and HuC/D in PBS-injected (n=5 for WT and n=5 *mmp9* mutant), NMDA-damaged (n=5 for WT and *mmp9* mutant, respectively), and light-damaged (n=8 for wt and *mmp9* mutant, respectively) retinas at 3DR.

a-d, and g-j data are presented as mean values +/- SEM. Asterisks indicate statistically significant differences between the indicated groups (**p* ≤ 0.05, ***p* ≤ 0.01, ****p* ≤ 0.001). Source data are provided as a Source Data file 1.


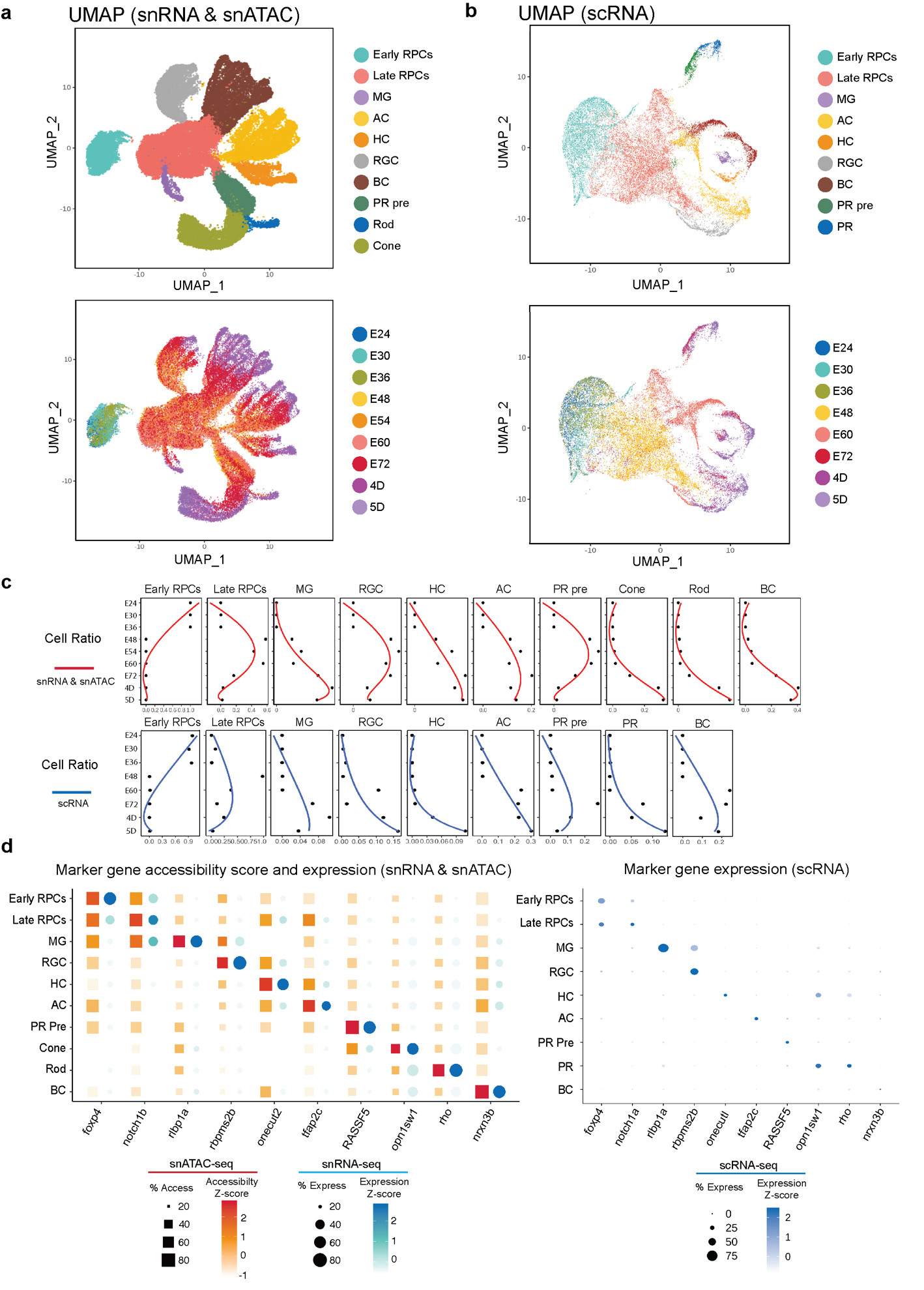


**Supplemental Figure 7: Overview of scRNA and snRNA/ATAC-Seq data from developing retina.**

(a) UMAP plots showing cells present in snRNA-Seq datasets from developing retina. Each point is colored by cell type (up panel) and time point (down panel).

(b) UMAP plots showing cells present in scRNA-Seq datasets from developing retina.  Each point is colored by cell type (up panel) and time point (down panel).

(c) The line plot (up panel:snRNA-Seq, down panel:scRNA-Seq) showing the changing of  cell ratios during the development. X-axis indicates the cell ratio and y-axis indicates each time point.

(d) Examples of mRNA levels and chromatin accessibility (left:snRNA-Seq and snATACseq, right:scRNA-Seq) for selected cell-type-specific genes.

**
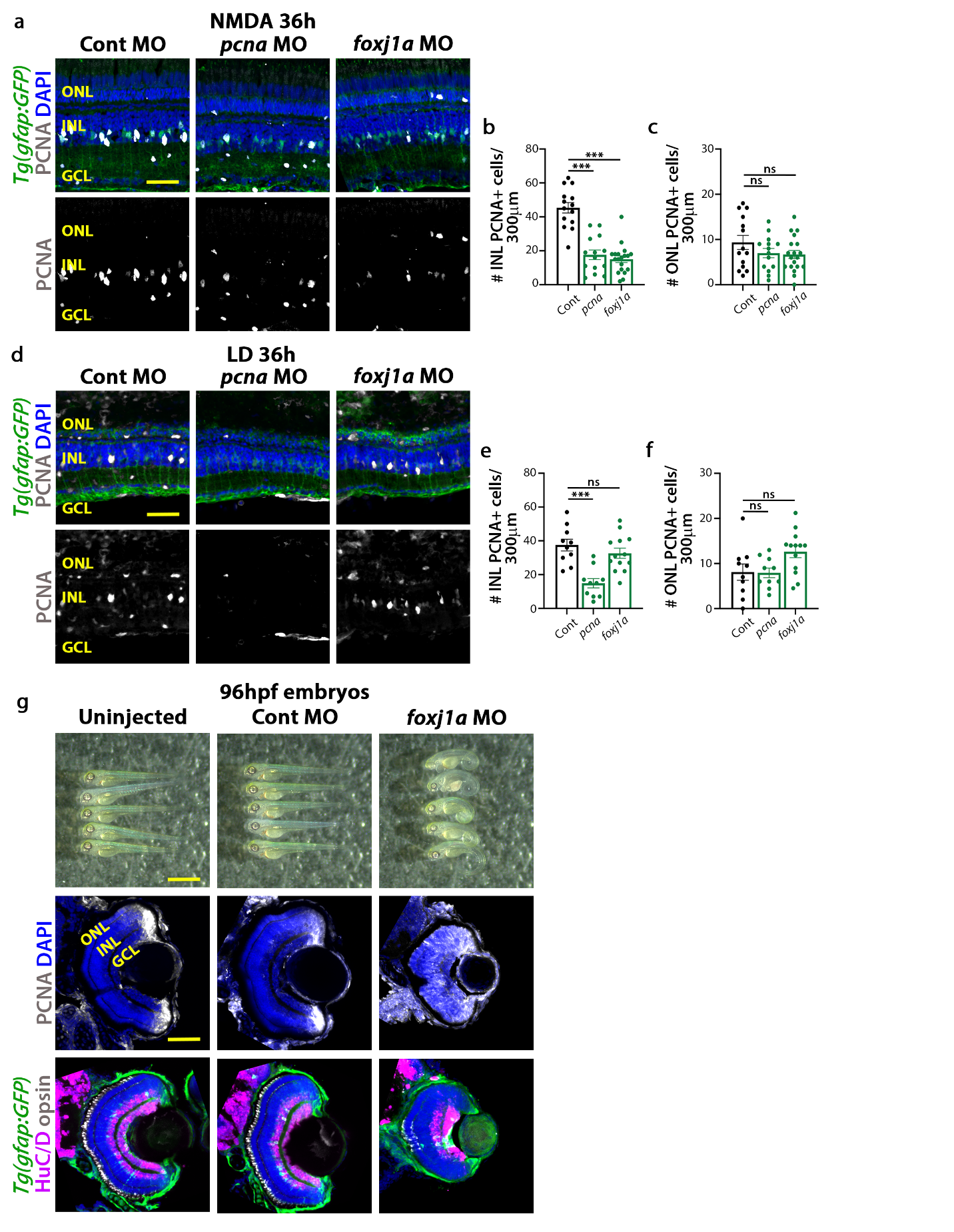
**

**Supplemental Figure 8: *foxj1a* is required for MGPC proliferation**.

(a) Tg(*gfap:GFP*) retinas electroporated with either Standard Control morpholino (Cont MO), *pcna* MO, or *foxj1a* MO were isolated 36 hours after NMDA injection and immunostained for PCNA, GFP, and counterstained with DAPI.

(b) Quantification of the number of PCNA-labeled cells in the INL. Cont n=14, *pcna* n=14, *foxj1a* n=19. Three independent experiments.

(c) Quantification of the number of PCNA-labeled cells in the ONL. Cont n=14, *pcna* n=14, *foxj1a* n=19. Three independent experiments.

(d) Tg(*gfap:GFP*) retinas electroporated with either Cont MO, *pcna* MO, or *foxj1a* MO were isolated 36 hours after starting constant light and immunostained for PCNA, GFP, and DAPI.

(e) Quantification of the number of PCNA-labeled cells in the INL. Cont and *pcna* n=10, *foxj1a* n=13. Three independent experiments.

(f) Quantification of the number of PCNA-labeled cells in the ONL. Cont and *pcna* n=10, *foxj1a* n=13. Three independent experiments.

(g) Uninjected and Standard Control morphants, as well as *foxj1a* morphants, were examined for gross morphological phenotypes at 96 hpf. Uninjected, Standard Control, and *foxj1a* morphant retinas were stained for PCNA and DAPI. In addition, *Tg(gfap:GFP)* uninjected, Standard Control, and *foxj1a* morphant retinas at 96 hpf were immunostained for HuC/D, green opsin (double cone photoreceptors), GFP, and counterstained with DAPI. Scale bars in a and d are 20μm, in g (top) is 1 millimeter, and in g (middle) is 50μm. b, c, e, and f data are presented as mean values +/- SEM. Asterisks indicate statistically significant differences between the indicated groups (****p* ≤ 0.001). Source data are provided as a Source Data file 1.
