# Supplementary material for: Common and divergent gene regulatory networks control injury-induced and developmental neurogenesis in zebrafish retina
Source: Nat Commun. 2023 Dec 20;14:8477. doi: 10.1038/s41467-023-44142-w (PMC10733277; doi:10.1038/s41467-023-44142-w)
Supplement: Supplementary file 3 — Description of Additional Supplementary Files [file 41467_2023_44142_MOESM3_ESM.pdf]

Description of Additional Supplementary Files

Supplemental Dataset 1: snRNA/ATAC-Seq and scRNA-Seq data for LD and NMDA samples.

Supplemental Dataset 2: Genes, peaks, and motifs selectively enriched in LD vs. NMDA samples (snRNA/ATAC-Seq+scRNA-Seq).

Supplemental Dataset 3: Regulatory relationships among TFs selectively active in LD vs. NMDA samples.

Supplemental Dataset 4: Genes selectively expressed in each of major retinal cell type in snRNA-seq and scRNA-Seq injury datasets.

Supplemental Dataset 5: snRNA/ATAC-Seq and reanalyzed scRNA-Seq data for developing retinal samples.

Supplemental Dataset 6: Genes, peaks, and motifs selectively enriched in injury vs. development samples (snRNA/ATAC-Seq+scRNA-Seq).

Supplemental Dataset 7: Regulatory relationships among TFs selectively active in injury vs. development samples.
